# Polycationic dendrimers synergizes with gefitinib to overcome EGFR^Ex19Del^-driven resistance in non-small-cell lung cancer

**DOI:** 10.1007/s12672-026-04934-0

**Published:** 2026-04-17

**Authors:** Adriana Cruz, Bruna Abreu, Cindy Mendes, Isabel Lemos, Catarina Freitas-Dias, Rafael Gomes, Bruna Sousa, Filipe Gonçalves, José S. Ramalho, Duarte C. Barral, Vasco D. B. Bonifácio, Jacinta Serpa

**Affiliations:** 1 iBB-Institute for Bioengineering and Biosciences, and Associate Laboratory i4HB-Institute for Health and Bioeconomy, Instituto Superior Técnico, Av. Rovisco Pais 1, Lisboa, 1049-001 Portugal; 2 iNOVA4Health, NOVA Medical School, Faculdade de Ciências Médicas, Universidade NOVA de Lisboa, Campo dos Mártires da Pátria, 130, Lisbon, 1169-056 Portugal; 3 Instituto Português de Oncologia de Lisboa Francisco Gentil (IPOLFG), Rua Prof Lima Basto, Lisboa, 1099-023 Portugal; 4 Bioengineering Department, Instituto Superior Técnico, Av Rovisco Pais 1, Lisboa, 1049-001 Portugal

**Keywords:** Non-small cell lung cancer (NSCLC), Tyrosine-kinase inhibitors, *EGFR* mutations, *KRAS* mutations, Polycationic polyurea (PURE) dendrimers, CAM

## Abstract

Lung cancer is a heterogeneous disease and the leading cause of cancer-related deaths worldwide. The aggressiveness of non-small-cell lung cancer (NSCLC) is often associated with oncogenic activation of the mitogen-activated protein kinase (MAPK) pathway. Among the most prevalent mutations in NSCLC, epidermal growth factor receptor 1 (EGFR) and Kirsten rat sarcoma (KRAS) alterations play a key role in cancer progression and patient survival. In this study, we investigated the influence of EGFR and KRAS mutational status on the response to polycationic core–shell dendrimers, using NSCLC cell lines harboring different EGFR and KRAS profiles. Cells were treated with PURE_G4_-OEI_48_ and PURE_G4_-OCEI_24_ dendrimers, which showed heterogeneous responses. In PC-9^*EGFREx19Del*^ cells, PURE_G4_-OEI_48_ induced elevated p-ERK/ERK ratios, indicating activation of the ERK-MAPK pathway and resistance. Notably, the EGFR^Ex19Del^ mutation contributed to this resistance. To overcome this effect, a synergistic strategy combining PURE_G4_-OEI_48_ and PURE_G4_-OCEI_24_ with the tyrosine kinase inhibitor (TKI) gefitinib was explored. This combination sensitized PC-9 cells, reducing ERK activation and enhancing cell death. *Ex vivo *chick chorioallantoic membrane (CAM) assays confirmed that EGFR^Ex19Del^ drives resistance to PURE_G4_-OEI_48_, whereas co-treatment with gefitinib improves efficacy. The use of complex dendrimer systems allows precise modulation of membrane-targeted interactions and intracellular signaling, providing mechanistic insights into overcoming EGFR^Ex19Del^-driven resistance. Overall, these findings highlight the translational potential of integrating membrane-targeted nanotherapeutics with EGFR-directed therapies to improve outcomes in NSCLC patients.

**Supplementary Information:**

The online version contains supplementary material available at 10.1007/s12672-026-04934-0.

## Introduction

Lung cancer (LC) is reported as the most incident type of cancer and a leading cause of death [1]. Among men, LC is the primary cause of both mortality and morbidity [1]. In women, the GLOBOCAN 2020 reported LC as the third most incident and the second mortality [2]. But the scenario has been changing with the GLOBOCAN 2022 report establishing LC as the second most common cancer in women [1]. Cigarette smoking is the major risk factor [3], however, the number of nonsmoking women diagnosed with LC has been increasing [4]. LC is a heterogeneous disease subdivided into two major groups of non-small-cell lung cancer (NSCLC) and small-cell lung cancer (SCLC). The latter type predominantly occurs in current or former smokers [5]. NSCLC accounts for 85% of the cases and presents a 5-year survival rate of 23% [6]. The main histological types included in NSCLC are the lung adenocarcinomas (LUAD), the squamous cell carcinomas (LUSC) and the large cell carcinomas. At the molecular level, oncogenic activation of the MAPK pathway is linked to NSCLC biology, primarily through mutations in Kirsten rat sarcoma (*KRAS*) [7] and mutations or overexpression of epidermal growth factor receptor 1 (*EGFR*) [8]. *EGFR* and *KRAS* are the predominant mutated genes in lung cancer [9]. The high NSCLC-related mortality rate is attributed to several factors: delayed diagnosis due to the absence of symptoms in early stages, molecular heterogeneity, which complicates the development of targeted treatments, and the emergence of resistance to chemotherapy and targeted therapies [2, 3]. Targeted and immune therapies brought a new hope to LC patients, but resistance mechanisms limit the efficacy of these strategies [10, 11]. Therefore, there is an urgent need for novel and effective therapeutic strategies [12, 13].

In this scope, dendrimers, a class of hyperbranched multivalent and monodisperse polymers, have gained attention over the past decades due to their ability to serve as nanovehicles for drug delivery, as dendriplexes to deliver genetic material, and as diagnostic tools in cancer [14, 15]. Polyurea (PURE) dendrimers have already been validated as effective nanocarriers in several cancer models, including NSCLC [15]. The next step is to exploit the dendrimers’ chemical properties to provide intrinsic anticancer potential [16, 17]. One strategy is to build dendrimers with a cationic structure [18–20] to more specifically target negatively charged cancer cells. It is widely reported that cancer cells reprogram their entire metabolism to proliferate and survive even in an unfavorable environment. This survival mechanism impacts multiple metabolic pathways, including lipid metabolism [21], which in turn affects the accumulation of lipid droplets [22] and the composition of the plasma and intracellular compartments’ membranes. Cancer cells lose the asymmetric composition characteristic of non-malignant cells, in which negatively charged lipids are maintained in the inner leaflet, by translocating phospholipids such as phosphatidylserine (PS) and phosphatidylethanolamine (PE) to the outer leaflet [23–26]. This phenomenon confers a negative charge to the plasma membrane, making it a potential target for novel dendrimer-based anticancer strategies.

As mentioned before, the development of EGFR tyrosine kinase inhibitors (TKIs) has represented a major therapeutic advance for patients with EGFR-mutated NSCLC. Despite the clinical benefits achieved with these targeted therapies, disease progression remains common due to the highly metastatic nature of NSCLC and the emergence of intrinsic and acquired resistance mechanisms [27]. Notably, EGFR-dependent signaling is closely associated with alterations in cell membrane dynamics, which may influence drug sensitivity and therapeutic response [28–30]. Therefore, we also investigated the impact of PURE dendrimers on NSCLC cell responsiveness to EGFR-targeted therapy, aiming to assess whether modulation of membrane properties could enhance the efficacy of EGFR inhibition and overcome resistance-associated phenotypes.

This study investigated PURE dendrimers, modified to have a positively charged core-shell structure, as therapeutic agents for NSCLC. The research emphasized the interference of *EGFR* mutations on PURE anticancer activity. We verified the EGFR^Ex19Del^ mutation drives resistance to PURE_G4_-OEI_48_, but combining it with gefitinib enhances cell death in NSCLC cells, highlighting the potential of integrating membrane-targeted and signaling-based therapies for better treatment outcomes.

## Materials and methods

### Preparation of polycationic core-shell PURE dendrimers

The synthesis of the fourth-generation polyurea dendrimer (PURE_G4_) was performed following our previous protocols [31, 32]. The POXylated intermediate PURE_G4_-OEtOx_48_, a PURE_G4_ dendrimer (core) surface conjugated with oligo-oxazolines (shell), was first prepared as previously described [33]. This core-shell intermediate dendrimer was used to prepare the final core-shell polycationic PURE dendrimers: a PURE_G4_ dendrimer with an oligoethyleneimine surface (PURE_G4_-OEI_48_), using our reported protocol [34], and a PURE_G4_ dendrimer with an oligochromyliumethyleneimine surface (PURE_G4_-OCEI_24_), prepared by a mechanosynthesis protocol published by our team [35]. This protocol was adapted from our previous work, where a polycationic oligo(ethyleneimine-*N*-chromylium salt) was prepared by reacting oligo(2-ethyl‐2‐oxazoline) (OEtOx, the PURE_G4_-OEtOx_48_ shell) with 2,4-dihydroxybenzaldehyde (2,4–DHB) and BF_3_.OEt_2_ using a conventional protocol [36].

Briefly, PURE_G4_-OEtOx_48_ (1.00 g, 0.0111 mmol), 2,4-DHB (1.08 g, 7.84 mmol) and HCl 37% (1.38 mL) were added to a 50 mL zirconium oxide reactor containing 150 zirconium oxide balls. The mixture was ground for 3 h at 500 rpm with rotation inversion cycles of 30 min (5-second pause between inversion cycles). After this period, 10 mL of methanol were added to the reactor. The methanolic solution was diluted with water and dialyzed for 24 h using a *SnakeSkin*™ dialysis tubing (3.5 K MWCO). The solid that precipitated inside the dialysis tube was filtered and recrystallized from methanol/diethyl ether. A red wine colored solid (0.67 g) was obtained in 95% yield. The molecular weight of PURE_G4_-OCEI_24_ was calculated by nuclear magnetic resonance (NMR), and only one chromylium chloride group per oligo-oxazoline chain was found to be formed, in only 24 oligo-oxazoline chains: *M*_w_= 63517.4 g/mol. ^1^H-NMR (300 MHz, DMSO-*d6*) δ (ppm): 8.62–7.94 (m, 96 H, ArH chromylium chloride, 24 chromylium chloride groups from the reaction of the *N*-acetyl units), 3.64 (m, PURE_G4_ core, partially overlapped with the water signal), 2.27 (1116 H, ArCH_3_ chromylium chloride from 24 groups + NCO*CH*_*2*_CH_3_ from 522 *N*-acetyl groups), 0.94 (1566 H, NCOCH_2_*CH*_*3*_ from 522 unreacted methyl groups from the *N*-acetyl units). The schematic structures is included in Supplementary Fig. 1.

### Cell culture

Human adenocarcinoma cell line A549 (CCL-185™, ATCC), H522 (CRL-5810™, ATCC) and H1975 (CRL-5908™, ATCC), adenosquamous carcinoma cell line H596 (HTB-178™, ATCC), and mucoepidermoid carcinoma cell line H292 (CRL-1848, ATCC) were obtained from American Type Culture Collection (ATCC, Manassas, VA, USA). The adenocarcinoma cell line PC-9 (90071810, ECACC) was obtained from European Collection of Authenticated Cell Cultures (ECACC, Porton Down, Salisbury, United Kingdom). Cells were cultured in Dulbecco’s Modified Eagle’s Medium 1× (DMEM) (41965-039, Gibco, Life Technologies) supplemented with 10% fetal bovine serum (FBS; S 0615, Merck), 1% Antibiotic-Antimycotic (AA; P06-07300, PAN Biotech) and 50 µg/mL Gentamicin (15750-060, Gibco, Life Technologies). Cells were maintained at 37 °C in a humidified environment with 5% CO_2_. Cells were cultured until an optical confluence of 75–100% and detachment was performed with 0.05% trypsin-EDTA 1 × (25300-054, Invitrogen). The NSCLC cell lines tested in this work presented distinct mutational profiles for *EGFR* and *KRAS* genes (Table 1).

**Table 1:** The NSCLC cell lines’ EGFR and KRAS gene mutational profiles

Cell line		
A549	*EGFR* ^WT^	*KRAS* c.34G > A (pG12S)
H292	*EGFR* ^WT^	*KRAS* WT
H522	*EGFR* ^WT^	*KRAS* WT
H596	*EGFR* ^amplification^	*KRAS* WT
H1975	*EGFR* ^L858R/T790M^	*KRAS* WT
PC-9	*EGFR* ^Ex19Del^	*KRAS* WT

### Cell death analysis by flow cytometry

Cells were seeded at a density of 1×⋅10^5^ cells/well in 24-well plates and cultured overnight under control conditions. The cells were then exposed to both polycationic core-shell dendrimers PURE_G4_-OEI_48_ and PURE_G4_-OCEI_24_ (0.38–51.2 µM) for 24 h. After the experimental conditions, cells in suspension in the conditioned media (supernatants) were collected, and adherent cells were detached with 0.05% Trypsin-EDTA. The cells in the supernatant and detached cells were harvested together by centrifugation, 255 × *g* for 2 min. Cells were stained with 0.5 µL annexin V fluorescein (FITC)-(640906, BioLegend, San Diego, CA, USA), in 1 × annexin V binding buffer (10 mM HEPES—pH 7.4, 150 mM NaCl, 2.5 mM CaCl_2_, prepared in 1 × PBS—pH 7.4), and incubated at room temperature, in the dark, for 15 min. Samples were then resuspended in 200 µL PBS 0.1% BSA and centrifuged at 255 × *g* for 2 min. The cells were resuspended in 200 µL of annexin V binding buffer 1 × and 1.25 µL of propidium iodide (PI, 50 µg/mL; P4170, Sigma-Aldrich; Darmstadt, Germany) was added 5 min before sample acquisition. Samples were acquired in a BD Accuri C6 Plus Flow Cytometer (Becton, Dickson and Company, Franklin Lakes, NJ, USA) and data analyzed using BD CSampler Software. Results were used to define the half-effect concentration (EC_50_) for both compounds in NSCLC cell lines. The gating strategy applied for subsequent analyses is schematized in Supplementary Fig. 2, together with representative dot plots from all controls used to properly define the quadrants. Briefly, cells were first gated to exclude cellular debris, followed by the exclusion of aggregates through singlet selection based on SSC-H versus SSC-A parameters.

To assess the effect of co-incubation with gefitinib and polycationic core-shell PURE dendrimers, 20 µM gefitinib (SML1657, Sigma-Aldrich, MO, USA) was tested alone and in combination with the other culture conditions.

### Zeta potential

Cells (2×10^5^ cells/well) were seeded in a 6-well plate to achieve 80% confluence within 24 h. Afterwards, A549, H292, and PC-9 cells were treated with PURE_G4_-OEI_48_ (25.15 µM) for 24 h. Then, cells were harvested using 0.05% Trypsin-EDTA. The detached cells were centrifuged (255 × *g* for 5 min) and washed with 1 × PBS. Before analysis with the Zetasizer Nano ZS, the cells were centrifuged again (255 × *g* for 3 min) and resuspended in ultrapure water. Dynamic light scattering measurements were performed using the Zetasizer Nano ZSP (Malvern Instruments Ltd, UK) in a Folded Capillary Zeta Cell (DTS1070, Malvern Instruments Ltd, UK) at 25 °C.

### Proliferation assay

Cell proliferation analysis was determined for H292 and PC-9 cells by generating proliferation curves. Cells were seeded in 24-well plates (5 × 10^4^ cells/well) in complete DMEM, synchronized under starvation and exposed to experimental conditions. Cells in suspension in the culture medium and adherent/trypsinized cells were counted at different time points (0, 6, 10, 24, 32 and 48 h) using a Burker cell counting chamber.

### Wound healing assay

The migratory capacity of the cells was examined by a wound healing assay. H292 and PC-9 cells were seeded in 12-well plates (2 × 10^5^ cells/well) with complete DMEM and maintained until 70–80% confluence. The cells were treated with Mitomycin-C (5 µg/mL, M4287, Sigma) for 3 h to inhibit cell division, and after this, the cell monolayer was scratched with a 200 µL pipette tip to create a wound. Cells were washed with PBS 1× to remove the suspended cells and afterwards maintained in experimental conditions. The leading edge of cell migration was monitored (Supplementary Fig. 3) by photography at 0, 6, 10, 24, 32 and 48 h using phase-contrast microscopy with the Olympus IX53 Inverted Microscope. Images were acquired and processed with Olympus *cellSens* software. *ImageJ* software (https://imagej.nih.gov) was used to analyze and quantify the wound closure.

### Western blotting

Cells were seeded (1×10^5^ cells/well) in 24-well plates and cultured under control conditions and exposed to 25.15 µM of PURE_G4_-OEI_48_ for 24 h. Total protein extracts were obtained after cell lysis in 20 mM MOPS (pH 6.5), 1% Triton X-100 (v/w) previously supplemented with 1 mM Na_3_VO_4_ (450243, Sigma Aldrich), 1 mM NaF (7681-49-4, Sigma Aldrich) and 1× protease inhibitors (11836170001, Roche). Protein concentration was determined by the Bradford method, using Bio-Rad protein assay reagent (500–0006, Bio-Rad) through spectrophotometric quantification (595 nm). After protein quantification, loading buffer containing 10% SDS, 0.5% bromophenol blue in Tris-HCL (pH 6.8) and 10% β-mercaptoethanol (M3148, Sigma) was added to 50 µg of the total protein and boiled at 95–100 °C for 10 min. Samples were loaded in a 12% Tris-glycine SDS-PAGE (Polyacrylamide gel), and electrophoresis was carried out in miniprotean Tetra Electrophoresis System (Bio-Rad). After, proteins were transferred to nitrocellulose membranes (Bio-Rad) with the Trans-Blot^®^ Turbo TM Blotting system. For protein detection, membranes were incubated with the primary specific antibodies anti-phospho-ERK 1/2 (1:1000, 9101, Cell Signalling) and anti-ERK 1/2 (1:1000, 137F5, Cell Signalling) at 4 °C overnight. Blots were further incubated with anti-rabbit horseradish peroxidase (HRP)-conjugated secondary antibody (1:5000, 31460, Thermo 42 Scientific) for 2 h at room temperature and immunoreactive bands were detected using the enhanced chemiluminescence (ECL) method in a ChemiDoc XRS System (Bio-Rad) with Image Lab software. As endogenous control, β-actin (mouse anti-human β-actin, 1:5000, A5441, Sigma) was used. Experiments were performed in biological triplicates.

### WST-8 assay for cell metabolic viability

Cell metabolic viability was assessed using Cell Counting Kit-8 (CCK-8; Dojindo Molecular Technologies, Gaithersburg, MD, USA) according to the manufacturer’s instructions. Cells were seeded in a 96-well clear bottom tissue culture plates (6 × 10^3^ cells/well/100 µL), and cultured under control conditions and exposed to 25.15 µM of PURE_G4_-OEI_48_ in the presence and absence of 20 µM of EGFR inhibitor gefitinib or 30 nM of MEK inhibitor trametinib (GSK1120212, S2673, Selleckchem), for 24 h. After this, cells were incubated with WST-8 reagent for 2 h at 37 °C and 5% CO_2_ saturation. The color was measure by spectrophotometric in an absorbance of 450 nm using a microplate reader (Bio-Rad, iMark 1681130).

### Passive diffusion assay

Cells (5×10^4^ cells/eppendorf) were used in the experiment performed at 37 °C and 4 °C to determine the contribution of passive diffusion and energy-dependent transport mechanisms. For cold conditions limiting active transport, H292, and PC-9 cells were placed at 4 °C during 30 min and then were treated with 25.15 µM PURE_G4_-OEI_48_ loaded with fluorescein (FL@ PURE_G4_-OEI_48_) with and without 20 µM of gefitinib for 60 min at 4 °C. For normal culture temperature allowing active transport, H292, and PC-9 cells cells were exposed to experimental treatment and maintained for 60 min at 37 °C. Samples were acquired in a BD Accuri C6 Plus Flow Cytometer (Becton, Dickson and Company, Franklin Lakes, NJ, USA) and data analyzed using BD CSampler Software. All experiments were conducted in biological triplicates.

### Immunofluorescence

Glass slides with a 0.2% gelatin coating (G-1890, Sigma Aldrich) were used to seed PC-9 cells (1 × 10^5^ cells/well) in 24-well plates, and cells were cultured under control conditions and exposed to PURE_G4_-OEI_48_ (25.15 µM) with and without gefitinib (20 µM), for 24 h. Cells fixation was performed with 4% paraformaldehyde for 15 min at 4 °C. Cells were fixed in 4% paraformaldehyde for 15 min at 4 °C. Following fixation, cells were incubated with 50 mM ammonium chloride (NH₄Cl) for 10 min. Blocking was performed using PBS 1× supplemented with 0.5% BSA and 0.1% saponin (w/v/v). Between each incubation step, cells were washed three times with PBS 1× for 5 min. After a 30 min blocking period, slides were incubated overnight at 4 °C with goat anti-human ATG12 antibody (AB0083, SICGEN; 1:100) diluted in PBS 1× − 0.5% BSA − 0.1% saponin (w/v/v). Slides were then rinsed and incubated for 2 h at room temperature with Alexa Fluor 488 donkey anti-goat secondary antibody (A-11055, Invitrogen, Thermo Fisher Scientific; 1:500) prepared in the same buffer. Slides were mounted with VECTASHIELD mounting medium containing DAPI (H-1200, Vector Labs, Newark, CA, USA) and examined under a Zeiss Imager.Z1 AX10 fluorescence microscope. Image acquisition and processing were performed using *CytoVision* software (https://www.leicabiosystems.com/pt-pt/ihc-ish/fish-molecular-solutions/cytogenetics/). The labelling quantification was performed with ImageJ software (imagej.nih.gov/ij/). All experiments were conducted in biological triplicates.

### Activity-dependent bulk endocytosis assay

PC-9 cells (1 × 10^5^ cells/well) were seeded on glass slides with a 0.2% gelatin coating (G-1890, Sigma Aldrich) in 24-well plates, and maintained under control conditions and exposed to PURE_G4_-OEI_48_ (25.15 µM) and gefitinib (20 µM), alone or in combination, for 2 h. After this time, 0.25 mg/mL Dextran Alexa Fluor Red (568) (Invitrogen) was added to each well and a pulse-chase experiment was performed using 0, 5, 10, 15 and 30 min timepoints. After incubation, cells were washed twice with 500 µL in 1⋅ PBS 0.1% BSA and centrifuged at 255 × *g* for 2 min, and samples were then resuspended in 200 µL in PBS 0.1% BSA for cytometry analysis. Samples were acquired in a BD Accuri C6 Plus Flow Cytometer (Becton, Dickson and Company, Franklin Lakes, NJ, USA) and data analyzed using BD *CSampler* software.

### *Ex vivo* chick chorioallantoic membrane (CAM) assay

The fertilized eggs were obtained from the hatchery *Pinto Valouro*, *Sociedade Agrícola da Quinta da Freiria*,* S.A.*, and incubated in a 40% humidified atmosphere at 37 °C. On day 3 of embryo development, a window was made into the eggshell, sealed with tape, and the eggs were returned to the incubator. On day 9 of embryo development, PC-9 cells (2 × 10^6^ cells) in 10 µL of Matrigel (Corning, 354230) were placed inside the eggs to allow the formation of a 3D tumor. On day 13 of development, four groups of tumors were established: the untreated; the treated with 20 µL of PURE_G4_-OEI_48_ (25.15 µM); the treated with gefitinib (20 µM), and the treated with PURE_G4_-OEI_48_ (25.15 µM) plus gefitinib (20 µM). After 96 h of treatment (corresponding to day 17 of embryo development), chicken embryos were euthanized by exposure to − 80 °C for 10 min. Tumor images were acquired digitally on days 13 and 17, and the *in ovo* tumor surface area was quantified using ImageJ software. The results were expressed as the change in tumor area between these two time points. For vascular assessment, *ex ovo* images were analyzed in ImageJ with the “Vessel Analysis” plugin, and data were presented as the percentage of the total area occupied by blood vessels. Following imaging, tumors were fixed in 4% paraformaldehyde at room temperature and subsequently embedded in paraffin for downstream analyses.

### Evaluation of *in vitro* hemolytic activity

Hemolysis was evaluated using the protocol described by Sæbø et al. [37]. In the scope of a collaboration with the Blood Service and Immune-Hemotherapy Department of *Instituto Português de Oncologia de Lisboa*,* Francisco Gentil*, peripheral blood samples from healthy donors were collected, under consent, in EDTA tubes and immediately centrifuged at 1700 × *g* for 5 min. The supernatant was removed by aspiration, and the erythrocyte *pellet* was washed three times with PBS. After the last washing step, the supernatant was discarded, and the *pellets* were diluted to a 1% erythrocyte suspension in PBS. The erythrocytes were plated in 96-well polypropylene plates with conical wells and treated with 50 µL of the conditioned media of PC-9 cells cultured in control conditions and exposed to PURE_G4_-OEI_48_ (25.15 µM) and gefitinib (20 µM), alone or in combination. After an incubation at 37 °C for 1 h, 2 h and 4 h, the plates were centrifuged at 1700 × *g* for 5 min. Lastly, 50 µL of the supernatants from each well were transferred into a flat-bottom 96-well plate. The absorbance of the supernatants was measured at 405 nm in an iMark Microplate Absorbance Reader (Bio-Rad). Triton X-100 (10%) was used as a positive control, and PBS as a negative control, in identical volumes as test compounds (50 µL).

### Tissue factor detection by Enzyme-Linked ImmunoSorbent Assay (ELISA)

Detection of extracellular concentration of the procoagulant protein Tissue factor (TF) was made by enzyme-linked immunosorbent assays (ELISA). Tissue factor is associated with hypercoagulation in cancer patients [38]. Extracellular TF was detected and quantified using the Human Tissue Factor ELISA kit (ab220653, abcam). Prior to the ELISA assays, the conditioned culture media of PC-9 cells cultured in control conditions and exposed to PURE_G4_-OEI_48_ (25.15 µM) and gefitinib (20 µM), alone or in combination, were concentrated in ultrafiltration centrifuge tubes (UFC8010, Merck, Sigma-Aldrich) at 3220 × *g* for 6 min. After concentrating the samples, the assays were performed following the manufacturer’s protocols. The original concentrations of TF in the tested samples were calculated by extrapolating the results measured to the original sample volume.

### Spectrophotometric determination of platelet aggregation

Platelets from multiple donors, suspended in plasma and additive solution for preservation were kindly provided by *Serviço de Imuno-Hemoterapia at Instituto Português de Oncologia de Lisboa Francisco Gentil* (IPOLFG) (IPOLFG-Ethical committee UIC-1349). The platelet suspensions were centrifuged at 2650 × *g* for 3 min. The platelets were treated with the conditioned culture media of PC-9 cells cultured in control conditions and exposed to PURE_G4_-OEI_48_ (25.15 µM) and gefitinib (20 µM), alone or in combination, and plated at a concentration of 5 × 10^5^ platelets/µL. Epinephrine was used as a positive control. The absorbance was measured after 5, 15, 30 and 60 min, at 595 nm with gentle shaking in an iMark Microplate Absorbance Reader (1681130, Bio-Rad). The relative aggregation for each experimental condition was calculated using the following formula:

$${\%\:Aggregation\:=\:}\frac{{\mathrm{Abs}}_{\mathrm{sample}}}{{\mathrm{Abs}}_{\mathrm{Pos}\mathrm{CTL}}}\times{100}$$

In which Abs _sample_ is the absorbance measured in the samples (platelets in conditioned culture media) and Abs _Pos CTL_ is the absorbance measured in the epinephrine positive control.

### Statistical and data analysis

All the results were plotted and analyzed in *GraphPad Prism* 8.0 software (www.graphpad.com). Data is presented as mean ± SD. Half-effect concentration (EC_50_) was calculated using the log inhibitor vs. response – variable slope (four parameter) method. Statistical analyses were also performed in *GraphPad Prism* 8.0 software (www.graphpad.com). Multiple comparisons between more than one group were performed using Two-way ANOVA with Sidak’s multiple comparisons test or One-way ANOVA with Tukey’s multiple comparisons test. Statistical significance was established at *p* < 0.05; * *p* < 0.05, ** *p* < 0.01, *** *p* < 0.001, **** *p* < 0.0001.

The synergy index for evaluating the combination of PURE_G4_-OEI_48_ and PURE_G4_-OCEI_28_ with gefitinib was calculated using the Bliss Independence model, in accordance with reported literature [39].

## Results

### PURE_G4_-OEI_48_ presented a higher anticancer potential compared to PURE_G4_-OCEI_24_

**Fig. 1:**
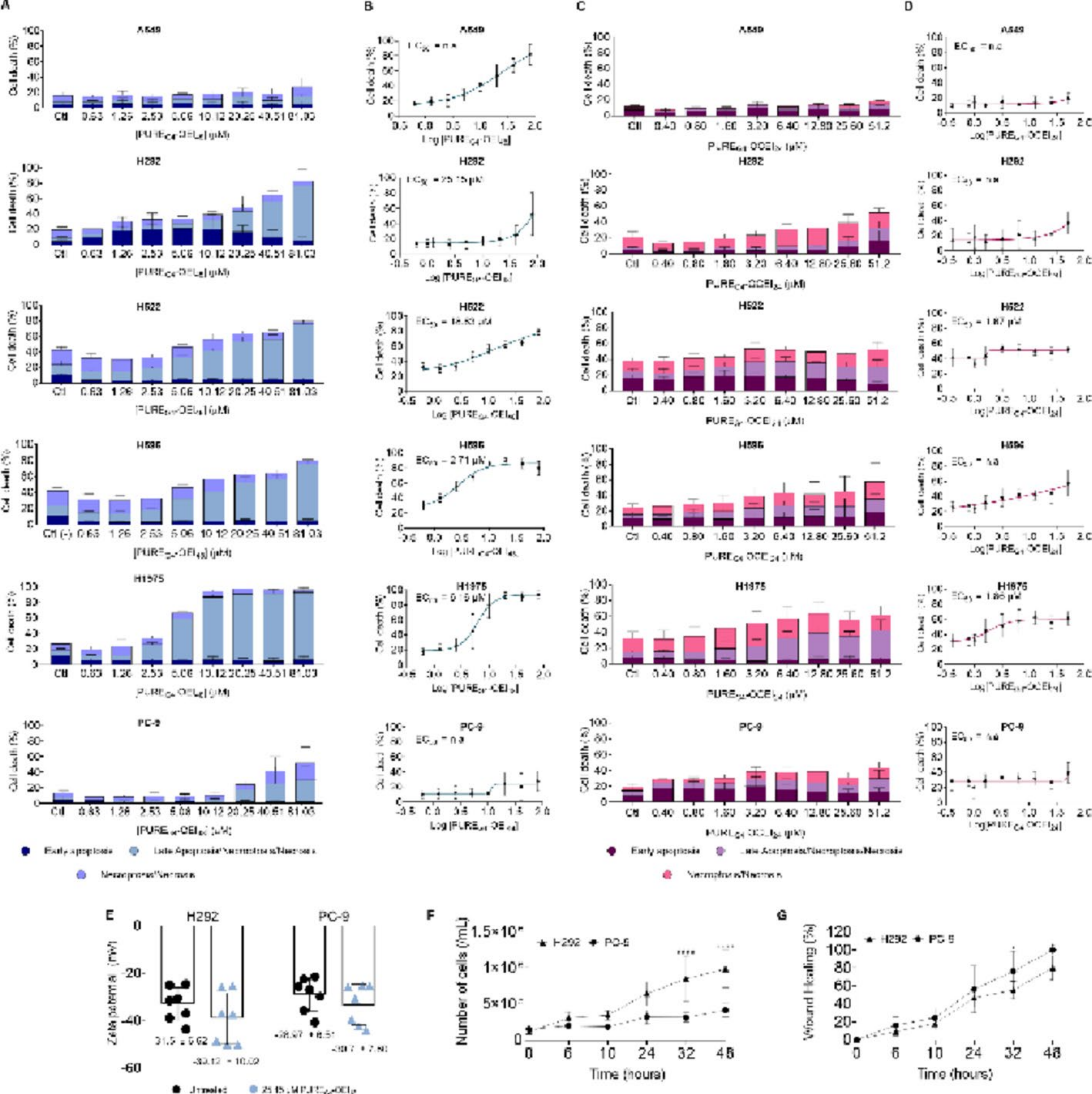
PURE_G4_-OEI_48_ showed a higher anticancer effect in NSCLC cells compared with PURE_G4_-OCEI_24_. Cell death was analyzed by flow cytometry using Annexin V-FITC and Propidium Iodide staining to distinguish cells in early apoptosis, late apoptosis/necroptosis/necrosis, and necroptosis/necrosis. Cell death levels and half-effect concentration (EC_50_) were determined in A549, H292, H522, H596 and PC-9 cells exposed to PURE_G4_-OEI_48_ **(A and B)** and PURE_G4_-OCEI_24_ **(C and D)**. The impact of PURE_G4_-OEI_48_ (25.15 µM) on cell polarization was evaluated in H292 and PC-9 cells by measuring the zeta potential **(E)**. The baseline proliferation **(F)** and migration **(G)** rates were determined in H292 and PC-9 cells cultured in control conditions to better characterize these cell lines before the treatment. All experiments were performed in biological triplicates and data are shown as mean ± SD, and *p < 0.01, ****p < 0.00001

To compare the anticancer effects of the two dendrimers, different NSCLC cell lines were exposed to both dendrimers for 24 h in different ranges of concentrations: PURE_G4_-OEI_48_ (0–81µM) and PURE_G4_-OCEI_24_ (0–52 µM). The NSCLC cell lines responded differently to both dendrimers, with PURE_G4_-OEI_48_ showing a higher anticancer potential against all cell lines (Figs. 1A and B) compared with PURE_G4_-OCEI_24_ (Figures C and D), except A549 and PC-9, with PC-9 cells being the least sensitive to PURE_G4_-OEI_48_. In contrast, PURE_G4_-OCEI_24_ dendrimer only induced cell death in H522 and H1975 cell lines (Figs. 1C and D). Regarding the cell death mechanisms induced by both dendrimers, cells treated with PURE_G4_-OEI_48_ exhibited a staining profile consistent with late apoptosis/necroptosis/necrosis, showing fluorescence for both Annexin V and PI. In NSCLC cells exposed to PURE_G4_-OEI_24_ (H522 and H1975), the cells were similarly distributed into late apoptosis/necroptosis/necrosis and necroptosis/necrosis.

### PURE_G4_-OEI_48_ tended to increase the zeta potential in H292 and PC-9 cells

To assess if PURE_G4_-OEI_48_ induces any alteration in membrane charge, the zeta potential of H292 and PC-9 cells was measured (Fig. 1E). Under control conditions, H292 cells exhibited a lower (more depolarized) zeta potential (−31.5 ± 6.62 mV) compared with PC-9 cells (−28.97 ± 6.51 mV). Because membrane polarization changes can be related to the functional state of the cells, the baseline proliferation and migration rates were assessed. H292 cells were found to be more proliferative than PC-9 cells (Fig. 1F), while PC-9 cells were more migratory than H292 cells (Fig. 1G). Upon exposure to PURE_G4_-OEI_48_, both cell types tended to decrease the zeta potential (Fig. 1E).

### p-ERK levels increase upon PURE_G4_-OEI_48_ exposure in the PC-9^*EGFR*Ex19Del^ cells, and gefitinib sensitizes these cells to PURE_G4_-OEI_48_

**Fig. 2:**
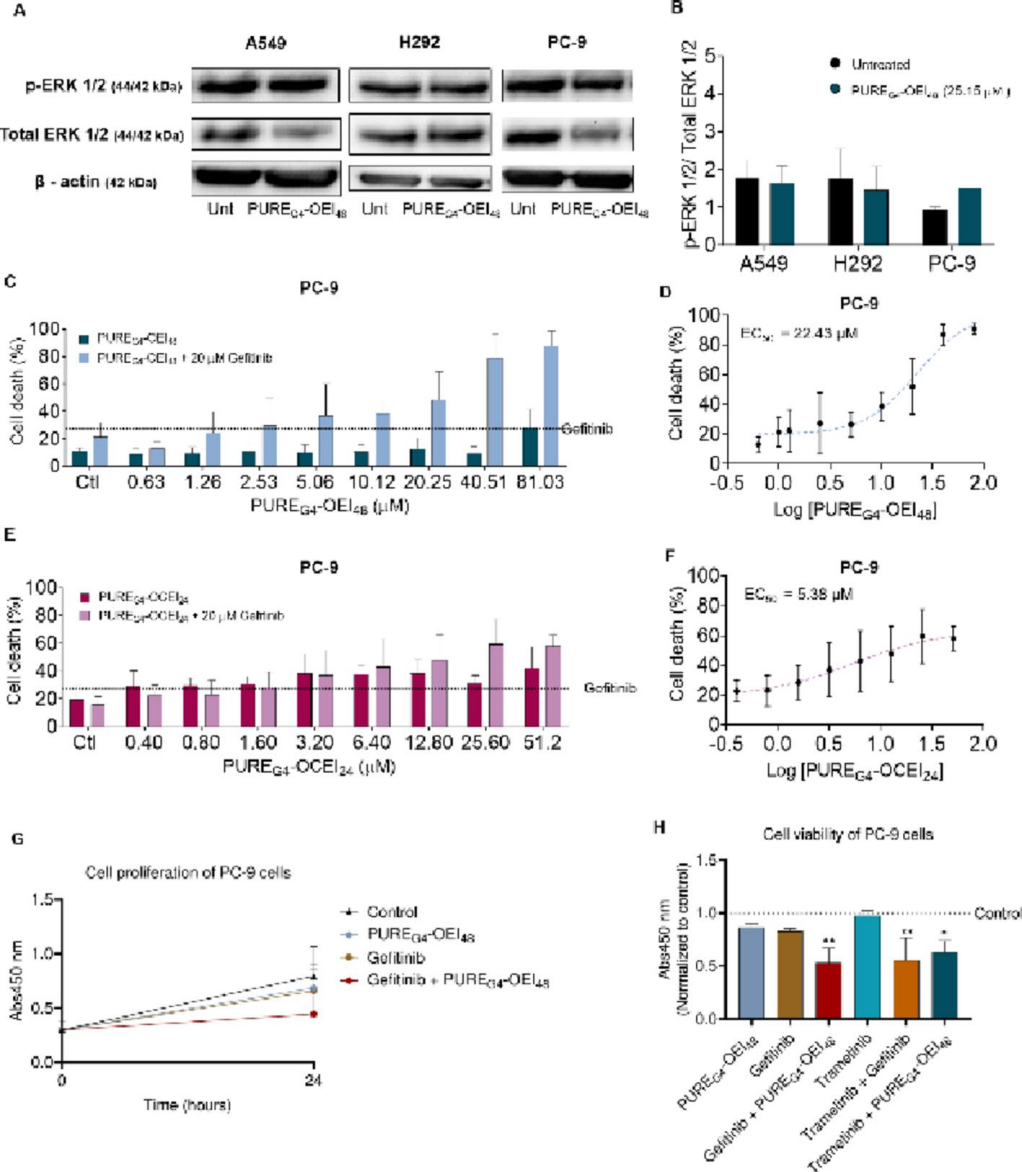
Gefitinib sensitizes PC-9 cells to PURE_G4_-OEI_48_ and PURE_G4_-OCEI_24_. Cells were cultured in control conditions and exposed to 25.15 µM of PURE_G4_-OEI_48_ for 24 h. The p-ERK 1/2 and total ERK1/2 were analyzed by western blotting, and α-tubulin was used as reference **(A)** and the ratio p-ERK1/2/ERK1/2 **(B)** was calculated using *ImageJ software*. The effect of EGFR inhibitor gefitinib in the sensitivity of PC-9 cells to PURE_G4_-OEI_48_ and PURE_G4_-OCEI_24_ was tested by measuring the cell death levels and EC_50_ in PC-9 cells exposed to PURE_G4_-OEI_48_ (0–81.2 µM) **(C and D)** and to PURE_G4_-OCEI_24_ (0–51.3 µM) **(E and F)** with 20 µM of gefitinib for 24 h. The line in C and E is related to cells exposed only to gefitinib. The 24 h-proliferation rate was measured with WST-8 metabolic viability assay in PC-9 cells exposed to PURE_G4_-OCEI_24_ (25.15 µM) with and without 20 µM of gefitinib **(G)**. The confirmation that the sensitizations of PC-9 cells to PURE_G4_-OEI_48_ effect was related to MAPK pathway was performed by WST-8 metabolic viability assay in cells exposed to PURE_G4_-OEI_48_ (25.15 µM) in the presence and absence of 20 µM of EGFR inhibitor gefitinib or 30 nM of MEK inhibitor trametinib **(H).** All experiments were performed in biological triplicates, except the dextran assay, and data are shown as mean ± SD, and *p < 0.01, **p < 0.001

To determine whether the impact of PURE_G4_-OEI_48_ on NSCLC was related to the oncogenic activation of the MAPK pathway, the expression levels of p-ERK and total ERK were analyzed (Figs. 2A and B) in NSCLC cell lines with *KRAS*^c.34G> A^ mutation (A549), *KRAS* ^wt^ and *EGFR*^wt^ profile (H292) and *EGFR*^Ex19Del^ mutation (PC-9) cells. Both A549 and H292 showed a trend to decrease p-ERK/ERK ratio upon PURE_G4_-OEI_48_ exposure, while PC-9 cells presented a trend to increase the p-ERK/ERK ratio after treatment, suggesting that the MAPK pathway was activated by PURE_G4_-OEI_48_.

Therefore, to confirm that the activation of MAPK pathway underlies the resistance to PURE_G4_-OEI_48_ in PC-9 cells, the EGFR inhibitor, gefitinib, was tested in combination with PURE_G4_-OEI_48_ and PURE_G4_-OCEI_24_. Gefitinib indeed sensitized PC-9 cells to dendrimers, and this was more evident in the increased cell death levels upon the combination of gefitinib with PURE_G4_-OEI_48_ than with PURE_G4_-OCEI_24_ (Figs. 2C, D, E and F). Accordingly, PC-9 cell exposed to gefitinib with PURE_G4_-OEI_48_ tend to proliferate less (Fig. 2G). These results suggest that gefitinib potentiates the PURE_G4_-OEI_48_ anticancer effect in cells bearing the *EGFR*^Ex19Del^ variant. Indeed, the synergistic interaction between gefitinib and the core–shell polycationic dendrimers in PC-9 cell lines was further validated by the synergy index (Supplementary Tables 1 and 2), calculated using the Bliss independence model. A synergy index greater than 0 denotes a synergistic effect between the two agents. As a functional confirmation of the involvement of MAPK pathway in the resistance of PC-9 cells to PURE_G4_-OEI_48_, cell metabolic viability was measured in cells exposed to MEK inhibitor in the absence and presence of PURE_G4_-OEI_48_. It was verified that MEK inhibition also sensitizes PC-9 cells to PURE_G4_-OEI_48_ toxicity, resulting in a loss of cell viability (Fig. 2H).

### Gefitinib potentiated endocytosis to facilitate PURE_G4_-OEI_48_ uptake in PC-9 cells

**Fig. 3:**
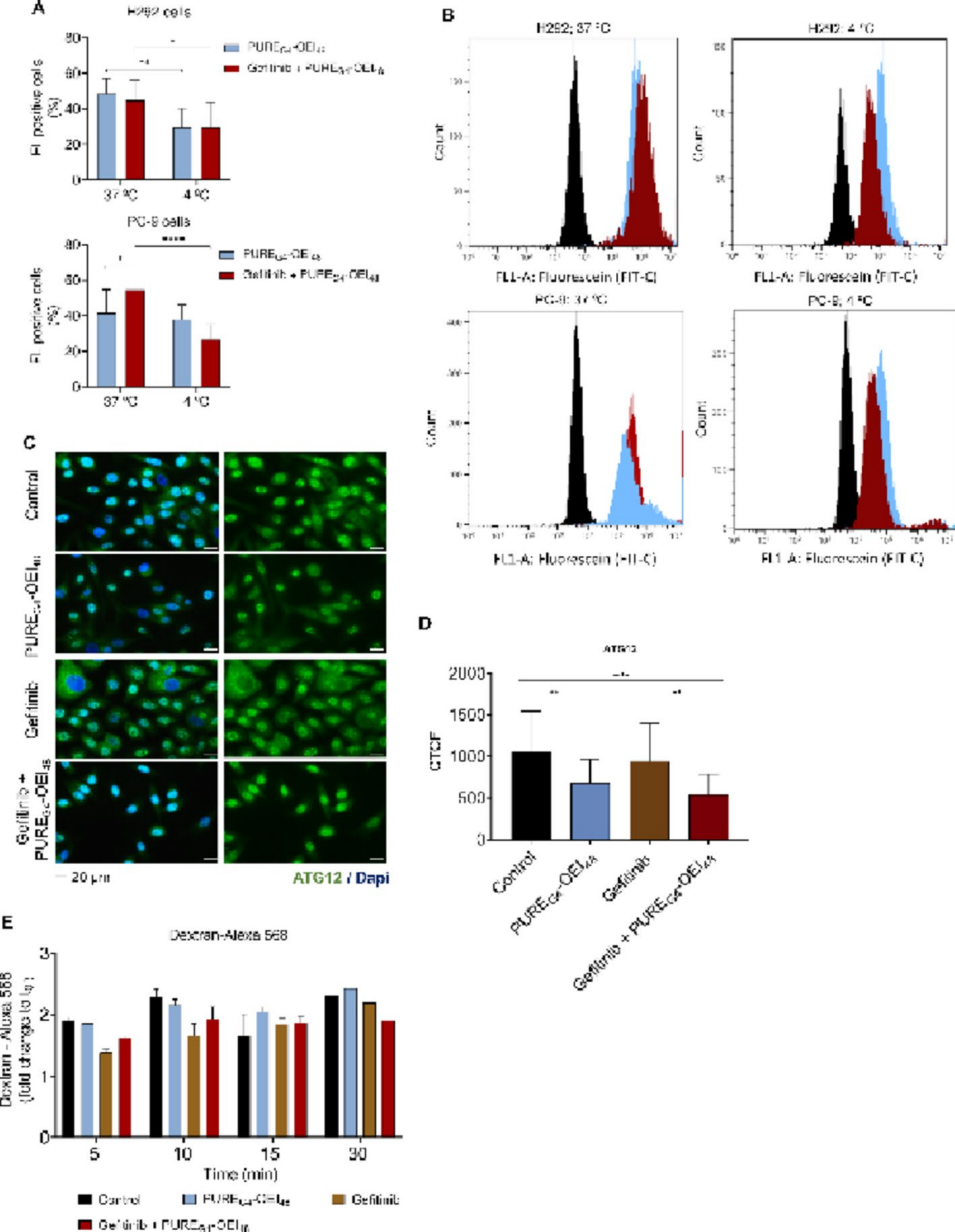
Gefitinib increases the contribution of active mechanisms mediating PURE_G4_-OEI_48_ uptake by PC-9 cells. The contribution of passive diffusion and active transport mechanisms accounting for uptake was evaluated by flow cytometry. Quantification of fluorescence in H292 and PC-9 cells exposed to 25.15 µM of fluorescein loaded PURE_G4_-OEI_48_ (FL@PURE_G4_-OEI_48_) in the presence or absence of 20 µM of gefitinib **(A)**. Representative histograms of flow cytometry **(B)**. Autophagy activation was assessed by ATG12 expression detected by immunofluorescence in **(C and D)** PC-9 cells exposed to PURE_G4_-OEI_48_ (25.15 µM) and of gefitinib (20 µM) in separate or in combination, for 24 h. Endocytosis was assessed by an activity-dependent bulk endocytosis assay, performed in a pulse-chase fashion (0, 5, 10, 15, 30 min) using dextran-Alexa 568 as a marker, in PC-9 cells exposed to PURE_G4_-OEI_48_ (25.15 µM) and to gefitinib (20 µM) in separate or in combination, for 2 h. Dextran levels were measured by **(E)** flow cytometry. All experiments were performed in biological triplicates, except the dextran assay, and data are shown as mean ± SD, and *p < 0.01, **p < 0.001, ****p < 0.00001

Given that PC-9 cells showed reduced sensitivity to PURE_G4_-OEI_48_ but became more responsive when combined with gefitinib, we explored whether passive diffusion and autophagy might play a role in this sensitization. In the passive diffusion assay, it was observed that gefitinib increased the uptake of FL@PURE_G4_-OEI_48_ in PC-9 cells but not in H292 cells at 37 °C (Fig. 3A and B). In the H292 cell line, FL@PURE_G4_-OEI_48_ uptake was significantly reduced at 4 °C compared with 37 °C. In contrast, in PC-9 cells, the temperature-dependent reduction was significant only upon exposure to FL@PURE_G4_-OEI_48_ plus gefitinib (Fig. 3A and B), suggesting that both passive diffusion and energy-dependent mechanisms mediate PURE_G4_-OEI_48_ entry into the cells. The significantly increased uptake at 37 °C and reduced uptake at 4 °C in the presence of gefitinib (Fig. 3A and B) indicate that gefitinib potentiates energy-dependent transport across the cell membrane.

To verify if autophagy could contribute to the loss of viability of PC-9 cells, the levels of ATG12, a macroautophagy marker [40], were measured by immunofluorescence in cells PURE_G4_-OEI_48_ alone or combined with gefitinib (Figs. 3C and D). PURE_G4_-OEI_48_ exposure significantly decreased ATG12 levels in PC-9 cells, regardless of gefitinib exposure (Figs. 3C and D). This observation suggests that PURE_G4_-OEI_48_ does not activate autophagy in PC-9 cells.

The effect of gefitinib on PURE_G4_-OEI_48_ uptake was evaluated through an activity-dependent bulk endocytosis assay performed by pulse-chasing dextran-Alexa 568, a fluid phase marker. We observed that after 5 and 10 min, dextran-Alexa 568 levels tended to be lower in cells exposed to gefitinib with or without PURE_G4_-OEI_48_, compared with control cells (Fig. 3E). At 15 min, no differences were observed between conditions, and after 30 min, cells exposed to gefitinib plus PURE_G4_-OEI_48_ showed decreased dextran levels compared with all the other conditions. Therefore, fluid phase uptake appears to be faster in cells treated with gefitinib, suggesting that gefitinib acts as an enhancer of endocytosis when combined with PURE_G4_-OEI_48_.

### Gefitinib combined with PURE_G4_-OEI_48_ significantly decreased the area of PC-9-derived tumors in the CAM model

**Fig. 4:**
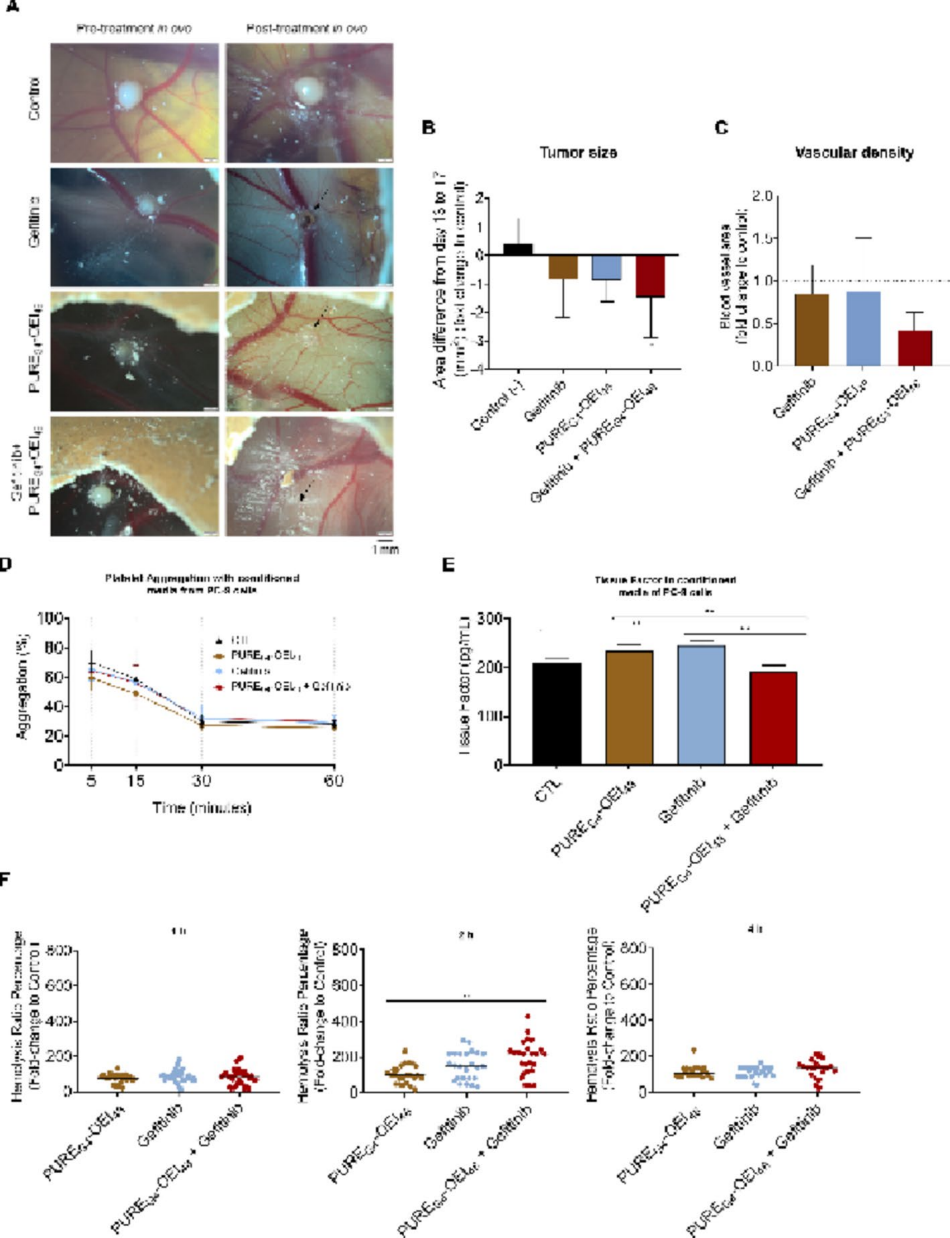
Gefitinib combined with PURE_G4_-OEI_48_ significantly tumor area in PC-9 derived tumors in the CAM model. Representative images of CAM-PC-9 derived tumors *in ovo* pre-treatment (day 13) and post-treatment (day 17) **(A)**. CAM-PC-9 derived tumors were maintained untreated to serve as a control and exposed to gefitinib (20 µM), PURE_G4_-OEI_48_ (25.15 µM) and gefitinib plus PURE_G4_-OEI_48_ for 4 days. The black arrow indicates the residual tumor. **(B)** The fold change of tumor area variation from day 13 to day 17, normalized to control tumors. **(C)** The vascular density, represented as the percentage of the total tissue area occupied by blood vessels, correlating vascular density and the vascular length density were calculated in *ImageJ* software using the Vessel Analysis plugin. **(D)** The effect on blood clot was explored by exposing platelets to the conditioned media of PC-9 cells cultured in control conditions and exposed to PURE_G4_-OEI_48_ (25.15 µM) and gefitinib (20 µM), alone or in combination and aggregation was measured after 5, 15, 30 and 60 min, at 595 nm. (**E**) Tissue factor was quantified in the conditioned media of PC-9 cells cultured in control conditions and exposed to PURE_G4_-OEI_48_ (25.15 µM) and gefitinib (20 µM), alone or in combination, by ELISA. (**F**) Hemolysis was followed for 1, 2 and 4 h by exposing whole blood to the conditioned media of PC-9 cells cultured in control conditions and exposed to PURE_G4_-OEI_48_ (25.15 µM) and gefitinib (20 µM), alone or in combination. All experiments were performed at least in biological triplicates, data are represented as mean ± SD, and *p < 0.01, **p < 0.001

To evaluate whether* ex vivo* gefitinib enhances PURE_G4_-OEI_48_ effect, CAM-PC-9-derived tumors were developed and treated (Fig. 4A). Gefitinib and PURE_G4_-OEI_48_, isolated or in combination, induced a decrease in tumor area. Significant differences in the fold change of variation in tumor area before (day 13) and 4 days after treatment (day 17) were observed in gefitinib and gefitinib plus PURE_G4_-OEI_48_ treated tumors compared with the control untreated tumors (Figs. 4A and B). Regarding vascular density, the percentage of the total tissue area occupied by blood vessels, correlating vascular density and vascular length density, was measured (Fig. 4C), and no differences were observed between the treated groups and the untreated tumors.

### The combination of PURE_G4_-OEI_48_ plus gefitinib did not affect platelet aggregation nor tissue factor (TF) production, however, it induced transient hemolysis

The overall concern on potential thromboembolic or vascular effects regarding the use of dendrimers as nanotherapeutics, we next examined platelet aggregation and TF production to evaluate whether the treatment could influence blood clot formation or coagulation. Regarding platelet aggregation, no statistical differences were observed between conditions, but PURE_G4_-OEI_48_ tended to decrease platelet aggregation, and this effect was rescued by gefitinib (Fig. 4D). The levels of TF were increased in conditioned media of PC-9 cells treated with PURE_G4_-OEI_48_ and gefitinib in separate; however, the difference was significant only for gefitinib exposure (Fig. 4E). Importantly, in the conditioned media of PC-9 cells exposed to the combination of PURE_G4_-OEI_48_ plus gefitinib, the levels of TF were similar to the control (Fig. 4E). Considering hemolysis induced by the treatment, no differences were observed at 1 h and 4 h of incubation, but at 2 h the conditioned media of PC-9 cells exposed to gefitinib tended to increase hemolysis rate, while the conditioned media of PC-9 cells treated with both PURE_G4_-OEI_48_ and gefitinib induced significantly increased hemolysis (Fig. 4F).

## Discussion

NSCLC accounts for 80–85% of all lung cancer cases, with a five-year survival rate of 15–23% [5, 6, 10]. Despite the emerging therapies, surgical resection is only an option for 25% of patients with early disease [41]. The discovery of driver mutations in oncogenes has been crucial for the design and development of targeted therapies for NSCLC. Herein, we disclosed PURE_G4_-OEI_48_ as a promising anticancer drug in NSCLC. Therefore, its administration, either isolated or in combination, deserves further investigation.

In this study, we confirmed the anticancer potential of polycationic core-shell dendrimer PURE_G4_-OEI_48_ against NSCLC cell lines in most cell lines tested, which are *EGFR* and *KRAS* wild type (Figs. 1A and B). The lower solubility of PURE_G4_-OCEI_24_ might explain its lower anticancer activity, compared with PURE_G4_-OEI_48_. Interestingly, in H292^*EGFRwt*/*KRAS*wt^, H522 ^*EGFRwt*/*KRAS*wt^, H596^EGFRAmp^ and H1975^*EGFR*L858R/T790M^ cell death was induced by PURE_G4_-OEI_48_ (Fig. 1A and B). While in A549^*KRASc.34G> A (pG12S)*^ and PC-9^*EGFREx19Del*^ cells, PURE_G4_-OEI_48_ was not efficient in inducing cell death. *KRAS* mutations are driving forces in resistance to EGFR inhibitors or immune checkpoints in NSCLC [42]. Moreover, *KRAS* mutations, except for KRAS^G12C^, are considered undruggable due to a lack of activity or selectivity [43, 44]. Regarding *EGFR*, exon 19 deletions, L858R substitution in exon 21 and T790M mutations promote the hyperactivation of downstream pro-survival signaling pathways [45], which are PI3K/mTOR [46] and MAPK [47]. *EGFR*-mutated tumors are treated with EGFR tyrosine kinase inhibitors (TKIs), which are typically efficient anticancer drugs. However, relapses due to acquired resistance often arise and are related to additional mutations, such as *EGFR* exon 20-T790 M, *HER2* and *MET* amplification, or mutations in *PI3KCA* and *BRAF* [48].

The improvement of PURE_G4_-OEI_48_ effect in PC-9 cells by the combination with gefitinib highlights the influence of the genetic background on the anticancer efficacy of polycationic core-shell PURE dendrimers (Fig. 2C). Activating *EGFR* mutations lead to persistent activation of the receptor even in the absence of ligand [49], favoring cell survival [50, 51]. PC-9 bears the Ex19Del *EGFR* activating mutation, which tended to increase the p-ERK1/2/ERK1/2 ratio upon exposure to PURE_G4_-OEI_48_. Therefore, cell death in PC-9 cells treated with PURE_G4_-OEI_48_ (Fig. 1A) was stimulated by the combination of gefitinib (Figures C and D). The underlying mechanism involves the MAPK pathway, as confirmed by the similar sensitization to PURE_G4_-OEI_48_ observed in PC-9 cells following inhibition of MEK (Fig. 2H), which is upstream of ERK1/2. Therefore, in EGFR^Ex19Del^ cells, such as PC-9, MEK inhibition is expected to decrease pERK1/2 [52, 53], reverting resistance to PURE_G4_-OEI_48_ and sensitizing PC-9 cells to PURE_G4_-OEI_48_ cytotoxic effects, resembling the effect of gefitinib. These results also indicate that PURE_G4_-OEI_48_ can improve the response of tumors to gefitinib, since the cell death levels were higher in cells exposed to the combination of PURE_G4_-OEI_48_ plus gefitinib compared with gefitinib alone (Fig. 2C).

In this study, it was confirmed that both passive diffusion and active transport mediate the uptake of FL@PURE_G4_-OEI_48_ by H292 and PC-9 cells (Figs. 3A and B), as fluorescence decreased at 4 °C compared with 37 °C in H292 and PC-9 cells exposed to FL@PURE_G4_-OEI_48_ alone or in combination with gefitinib. Exposure to low temperatures such as 4 °C has been shown to impair mitochondrial ATP synthase activity and oxidative phosphorylation, leading to reduced ATP production in cells. This energetic deficit compromises ATP-dependent transport systems, as their function depends on sufficient cellular ATP supply for active translocation across membrane [54–56]. Moreover, the increased fluorescence at 37 °C in PC-9 cells exposed to PURE_G4_-OEI_48_ plus gefitinib, compared with cells exposed to FL@PURE_G4_-OEI_48_ alone, indicates that gefitinib enhances the uptake of FL@PURE_G4_-OEI_48_, specifically increasing the contribution of active transport (Fig. 3A and B). Additionally, the significant reduction of fluorescence in PC-9 cells exposed to FL@PURE_G4_-OEI_48_ plus gefitinib at 4 °C compared with 37 °C, indicates gefitinib preferentially stimulates active transport (Fig. 3A and B). Importantly, gefitinib only affects FL@PURE_G4_-OEI_48_ uptake in PC-9 EGFR^Ex19Del^ cells and not in H292 EGFR^wt^ cells. Interestingly, it was demonstrated that TKI-sensitive cells, such as PC-9 cells, present an adaptive endosomal behavior to allows EGFR recycling back to the plasma membrane, trafficking instead through Rab7-positive late endosomes to lead to mutant EGFR degradation [57]. Additionally, PURE_G4_-OEI_48_ does not seem to activate autophagy, as indicated by the decreased levels of ATG12 in cells exposed to PURE_G4_-OEI_48_ with or without gefitinib (Figs. 3C and D). ATG12 is essential for autophagosome maturation, a process involved in macroautophagy, which is a conserved and complex intracellular degradative pathway [58]. In is reported that upon gefitinib, TKI-resistant NSCLC cell lines hyperactivates autophagy and EGFR colocalizes with LC3, the same is not observed in TKI-sensitive NSCLC cell lines, ibncluding PC-9 [57]. Although, ATG12, together with ATG3, regulates basal autophagy and endolysosomal trafficking [59], ATG12 is required for late endosome distribution [60]. Therefore, if PURE_G4_-OEI_48_ is entering the cell through endocytosis, the decreased levels of ATG12 may be related to endolysosomal-associated degradation. Although further studies are required, the reduced levels of dextran-Alexa 568 over time in cells exposed to gefitinib, with or without PURE_G4_-OEI_48_ (Fig. 3E), suggest an increase in endocytic activity. Nevertheless, gefitinib-induced EGFR reduction is possibly related to endocytic trafficking through Rab7-positive late endosomes autophagosome-mediated EGFR degradation [57]. Dextran enters the cells through fluid-phase (bulk) endocytosis [61], and as mentioned previously, endocytosis and autophagy share some processes and features [62]. However, an unclear issue is the balance between bulk conversion through compartment fusion and the precise contribution of autophagy and endocytosis, which is influenced by vesicle trafficking and fusion [63]. An intricate circuit of events interconnects the different types of endocytosis [64], often complicating the characterization of the uptake of new drugs. It is described that receptor-mediated endocytosis, through clathrin-coated vesicles, cooperates with bulk endocytosis [64, 65], reinforcing the possibility that EGFR-mediated endocytosis may enhance dextran uptake. Since gefitinib can activate autophagy to degrade mutant EGFR, and autophagy is also linked to bulk endocytosis, it is likely that gefitinib accelerates dextran uptake and degradation in PC-9 cells. Therefore, this study highlights a complex network of events that regulate endocytosis and autophagy, warranting deeper exploration in the future.

Furthermore, the endocytosis and exocytosis dynamics influence and are influenced by the plasma membrane composition and charge [66, 67], putatively affecting the efficacy of PURE_G4_-OEI_48_. Therefore, gefitinib, by efficiently blocking EGFR^Ex19Del^ [45], may disrupt the trafficking flow and improve the sensitivity to PURE_G4_-OEI_48_. This was validated in a CAM assay, in which the *ex vivo* anticancer efficacy of gefitinib and PURE_G4_-OEI_48_ combined strategy was tested (Fig. 4). Consistent with our *in vitro* findings, gefitinib sensitized CAM-PC-9-derived tumors to PURE_G4_-OEI_48,_ resulting in a drastic reduction in the variation of tumor area from day 13 and day 17 (Fig. 4B). While both gefitinib and PURE_G4_-OEI_48_ reduced tumor area, when administered individually, gefitinib was more efficient, which is in line with the efficacy of TKIs against *EGFR*-mutated NSCLC cells [45]. In agreement with the *in vitro* studies, PURE_G4_-OEI_48_ treatment showed no significant alteration in tumor area (Fig. 4B), reinforcing that *EGFR* mutations, namely *EGFR*^Ex19Del^ mutation, may confer resistance to the polycationic core-shell PURE dendrimers. This is confirmed by the observation that tumors treated with PURE_G4_-OEI_48_ plus gefitinib have a significant reduction in tumor area (Fig. 4B). In terms of angiogenesis, no antiangiogenic response was observed in any treatment group, considering the vascular density levels (Fig. 4C). However, PURE_G4_-OEI_48_ plus gefitinib showed a trend to reduced vascularization. Positively charged nanoparticles have been described as angiogenesis promoters, with the capacity of activating endothelial cells *in vitro* [68], but in our study, no alterations in vessel density were observed due to PURE_G4_-OEI_48_. Overall, the *ex vivo* assay results are promising, and reinforced the CAM assay is a cost-effective and efficient method for screening novel drugs, as it enables rapid tumor cell xenografting and the observation of both tumor growth and angiogenesis [69].

The polycationic core-shell PURE dendrimers have not been explored in the context of vascular alterations and thromboembolic events. Given that poly(amidoamine) (PAMAM) dendrimers and other nanoparticles have been reported to induce blood clots *in vitro* and in animal models [70, 71], and considering that most cancer patients already have an elevated risk of thromboembolism [72–74], a therapeutic approach that further promotes blood clotting is undesirable. Although, the effect of PURE_G4_-OEI_48_ on blood clot formation and coagulation should be explored in the future, so far, we have found no alterations in platelet aggregation by PURE_G4_-OEI_48_ and gefitinib alone or in combination (Fig. 4D), but, importantly TF expression is stimulated by gefitinib and reverted by PURE_G4_-OEI_48_ (Fig. 4E). This observation suggests PURE_G4_-OEI_48_ as an alternative to prevent thromboembolic issues related to gefitinib [75, 76]. Regarding hemolysis, the significant increase upon 2 h of exposure to PURE_G4_-OEI_48_ plus gefitinib was solved after 4 h of treatment, which suggests hemolysis may be a transient issue (Fig. 4F). However, more (patho)physiological in vivo assays, such as murine cancer models, must be performed to validate the efficacy and address side/adverse effects of the gefitinib and PURE_G4_-OEI_48_ combinatory strategy. Moreover, in a translational setting, PURE_G4_-OEI_48_ and PURE_G4_-OCEI_24_ could also be administered as dry powder via inhalation, rather than endovenously. Recently, our team reported a strategy employing lactic acid–functionalized PURE dendrimers loaded with selenium–chrysin, which, when administered by inhalation, significantly reduced NSCLC tumor size in mice [77]. Regarding toxicity, which is a critical aspect for the translational potential of these compounds, *in vitro* studies using non-malignant cell lines have previously shown that both core–shell polycationic dendrimers do not induce significant cellular damage [35, 78]. These findings suggest a favorable safety profile at the cellular level. In addition, the in vivo toxicity of these dendrimers has already been investigated by our group using murine models of triple-negative breast cancer [78]. In that study, systemic toxicity was assessed by measuring serum biomarkers associated with neural, hepatic, renal, and cardiac toxicity. The results indicated that, overall, both dendrimers exhibited low toxicity, with the exception of hepatic effects, as PURE_G4_-OCEI_24_ was associated with increased levels of aspartate aminotransferase. Despite this observation, no significant alterations were detected in the other evaluated toxicity markers.

Interestingly, by measuring the zeta potential (mV) in PC-9^*EGFR*Ex19Del^ and H292^*EGFRwt*/*KRAS*wt^ cells upon PURE_G4_-OEI_48_ treatment revealed an impact on zeta potential values (Fig. 1E). H292 cells clearly showed a lower zeta potential that can be related to an increase in PS residues in apoptotic cell membranes [79]. The higher zeta potential in PC-9 cell membrane may be attributed to an increase in PS residues on the membrane, though not in a significant quantity detectable by Annexin V. The exposure of PS in the outer leaflet may occur in other situations as a response to cellular stress, namely as a consequence of autophagy in which the PS translocation may occur [80]. In control conditions, zeta potential values *EGFR*^*mut*^ cells are higher than *EGFR*^*wt.*^ Membrane and zeta potentials regulate biological processes such as proliferation, with depolarized cells (lower zeta potential) tending to be more proliferative [81, 82]. Accordingly, H292 cells showed higher proliferative rates compared to PC-9 cells (Fig. 1F). In contrast, the migratory capacity was higher for PC-9 (Fig. 1G). It must be noted that cell proliferation and migration are asynchronous processes that depend on multiple cellular pathways beyond those regulated by EGFR [83].

## Conclusion

Overall, our study uncovers the heterogeneity of NSCLC and its distinct responses to polycationic dendrimers, highlighting PURE_G4_-OEI_48_ as a promising tool for developing novel therapeutic strategies. The application of PURE_G4_-OEI_48_ may be adjusted to the NSCLC genetic background, and its applied combination with other drugs could help overcome resistance mechanisms. In this line, we demonstrated *EGFR*^Ex19Del^ mutation underlies resistance to PURE_G4_-OEI_48,_ and the combination of gefitinib with PURE_G4_-OEI_48_ synergistically contributes to improving the induction of cell death in NSCLC cells. These results reinforce that integrating membrane-targeted strategies with existing cell signaling-based targeted therapy could improve NSCLC treatment outcomes. In terms of translational feasibility, polyurea dendrimers offer several advantages, including a well-defined molecular composition, reproducible synthesis, and customizable surface chemistry. These characteristics are essential for scalability and regulatory evaluation. Furthermore, polycationic dendrimers and related architectures are already being explored in preclinical and clinical settings, further supporting their potential for translational relevance.

## Supplementary Information

Supplementary Material 1

## Data Availability

The datasets generated during the current study are available on the public repository GitHub (https://github.com/lgafeira/LungCancerResearch).
