# Supplementary material for: Polycationic dendrimers synergizes with gefitinib to overcome EGFREx19Del-driven resistance in non-small-cell lung cancer
Source: Discov Oncol. 2026 Apr 17;17:811. doi: 10.1007/s12672-026-04934-0 (PMC13216407; doi:10.1007/s12672-026-04934-0)
Supplement: Supplementary file 1 — Supplementary Material 1 [file 12672_2026_4934_MOESM1_ESM.pdf]

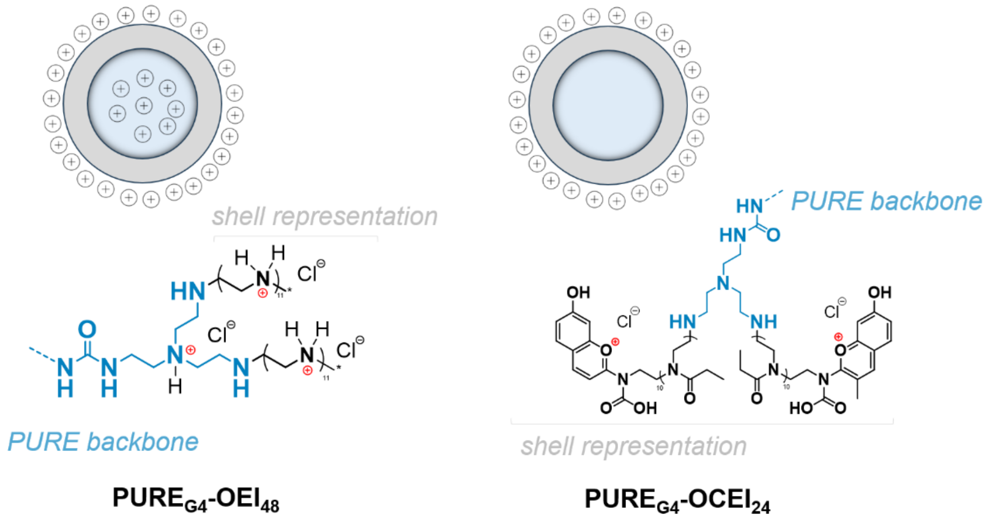

Supplementary Figure 1. Schematic representation of core-shell polycationic PURE dendrimers PURE$_{G4}$-OEI$_{48}$ and PURE$_{G4}$-OCEI$_{24}$.

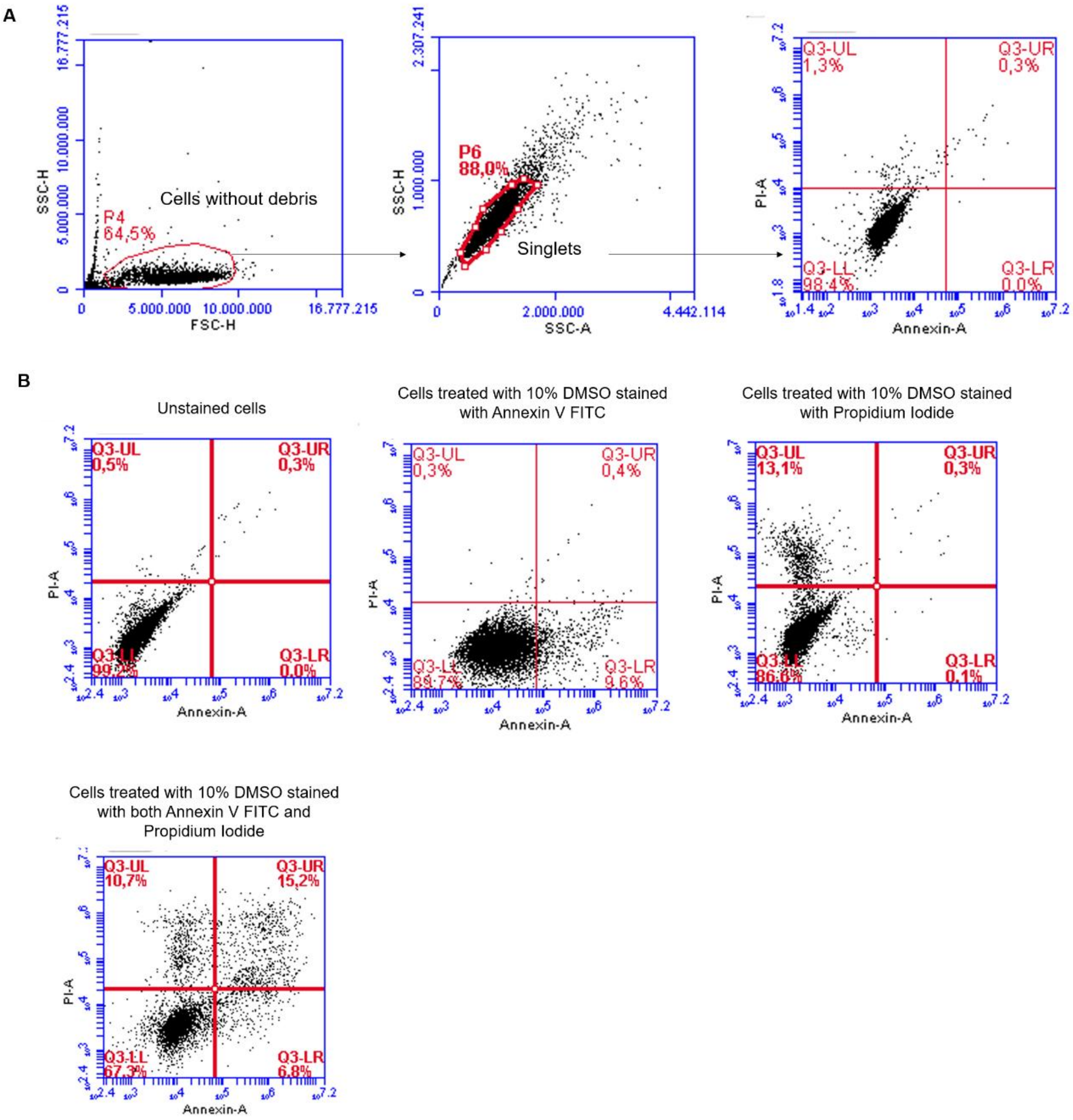

Supplementary Figure 2. Representative dot plots from flow cytometry analysis of the H292 cell line. (**A**) Gating strategy applied to all samples: cells were first grouped to exclude debris, followed by singlet selection to remove cellular aggregates. Using this final gate, different controls were analyzed to better define the quadrants. (**B**) Various controls used in this experimental setup: unstained cells, cells treated with 10% DMSO stained only with Annexin V FITC or propidium iodide (PI), and cells stained with both dyes.

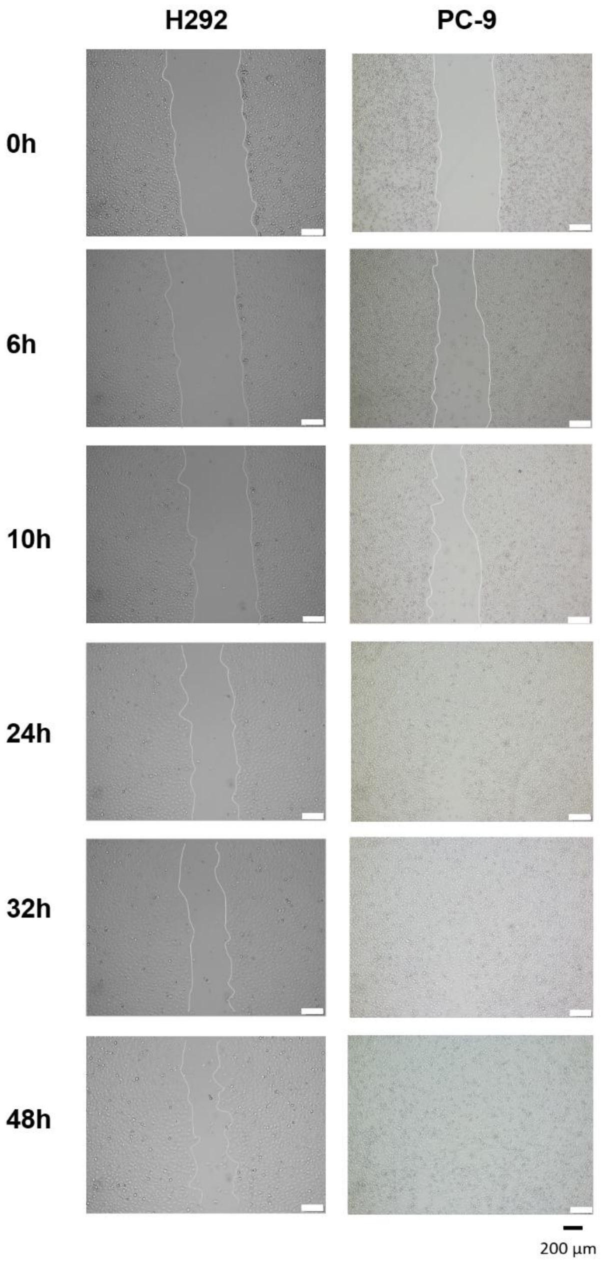

Supplementary Figure 3. PC-9 cells exhibit higher migratory capacity than H292 cells. Migratory capacity of NSCLC H292 and PC-9 cells was assessed at different time points. Wound closure was quantified over time by measuring the region of interest (ROI), as illustrated in the schematic above. The analysis was performed using ImageJ software. All biological experiments were carried out in triplicates for each time point.

Supplementary Table 1. Synergy index calculated for the combination of gefitinib and $PURE_{G4}$-OEI_{48} using the Bliss independence model.

**[$PURE_{G4}$-OEI_{48}], μM**	**Synergic index**
0.63	-0.20
1.26	0.18
2.53	0.22
5.06	0.29
10.12	0.31
20.25	0.39
40.51	0.72
81.03	0.67

Supplementary Table 2._Synergy index calculated for the combination of gefitinib and $PURE_{G4}$-$OCEI_{24}$ using the Bliss independence model.

**[$PURE_{G4}$-$OCEI_{24}$], μM**	**Synergic index**
0.40	0.02
0.80	0.02
1.60	0.06
3.20	0.09
6.40	0.17
12.80	0.20
25.60	0.37
51.2	0.27
